# Two-Dimensional CeO_2_/RGO Composite-Modified Separator for Lithium/Sulfur Batteries

**DOI:** 10.1186/s11671-018-2798-5

**Published:** 2018-11-23

**Authors:** Suyu Wang, Fan Gao, Yan Zhao, Ning Liu, Taizhe Tan, Xin Wang

**Affiliations:** 1 School of Materials Science and Engineering, Hebei University of Technology, Tianjin, 300130 China; 2 Synergy Innovation Institute of GDUT, Heyuan, 517000 China; 3 International Academy of Optoelectronics at Zhaoqing, South China Normal University, Guangzhou, Guangdong Province China

**Keywords:** CeO_2_/RGO composite, Modified separator, Lithium/sulfur batteries

## Abstract

In this work, a modified separator coated with a functional layer of reduced graphene oxide (RGO) anchored by cerium oxide (CeO_2_) nanoparticles was developed. The superior conductivity of RGO and chemical immobilization of high-ordered sulfur-related species (mainly Li_2_S_*n*_ 4 ≤ *n* ≤ 8) of CeO_2_ yielded batteries with enhanced characteristics. A remarkable original capacity of 1136 mAh g^−1^ was obtained at 0.1 C with capacity retention ratio of 75.7% after 100 charge/discharge cycles. Overall, these data indicate that the separator with CeO_2_/RGO composite is promising to suppress the shuttling of polysulfides for better utilization of the active material.

## Background

High-performance rechargeable batteries are currently being developed to meet the urgent demands of high-specific capacity and superior energy density application devices. Li/S batteries have widely been considered as promising energy storage for power grids and electric devices because of their outstanding theoretical capacity (1672 mAh g^−1^) and prominent energy density (2600 Wh kg^−1^) [1, 2]. However, despite their numerous advantages, some major obstacles hindering their practical commercial usage of Li/S batteries are still to be solved. Firstly, the insulating nature of the active material (S_8_) and its discharge products (Li_2_S_2_/Li_2_S) can cause poor electrochemical accessibility and decrease utilization of active materials [3, 4]. Secondly, polysulfides tend to dissolve in organic electrolytes after numerous charge/discharge processes and readily diffuse across the separator to be finally reduced to Li_2_S_2_ or Li_2_S solids at the surface of counter electrode. This results in low coulombic efficiency and poor cycling life of Li/S batteries [5, 6].

Tremendous efforts have been made to improve the conductivity and deal with the shuttling of polysulfides. These include modification of sulfur cathodes by confining more sulfur into porous conductive frameworks [7], implementation of a functional interlayer between the cathode and separator as polysulfides host [8, 9], and optimization of organic electrolytes [10]. However, recent studies have shown that the diffusion of polysulfides is difficult to fully overcome. Considering that, the newly developed methods used to improve the performance of Li/S batteries by modifying the separator have attracted increasing attention. For example, materials like functional carbon [11], graphene [12], active carbon [13], polypyrrole [14], and various metal oxides [15] have been used as coatings for the separator or as free-standing interlayers. These functional components would inhibit the migration of sulfur-related species to the anode and improve the electrical conductivity of cathodes. Studies have shown that reduced graphene oxide (RGO) interlayer might reduce the charge-transfer resistance (*R*_CT_) of sulfur cathodes while acting as an upper-current collector [16]. The latter is related to the improved utilization of sulfur. On the other hand, metal oxides like Al_2_O_3_ [17], MgO [18], NiFe_2_O_4_ [19], and SiO_2_ [20] can absorb polysulfides by introducing strong chemical bonds. However, the added interlayer may increase the total mass of the cell, resulting in declined energy density.

**Fig. 1:**
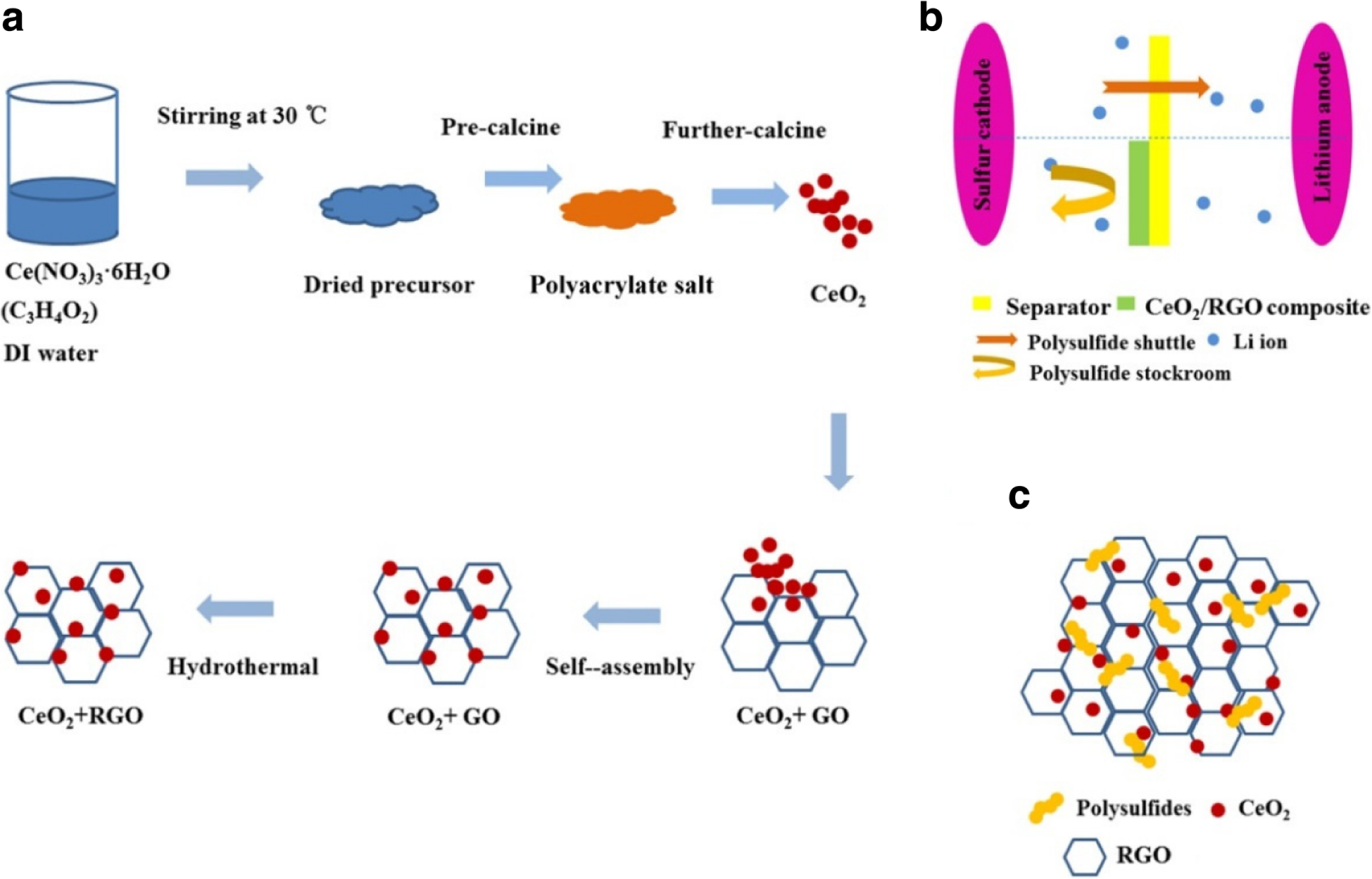
A schematic illustration of fabrication process of CeO_2_/RGO composite (**a**). Scheme of the cell configuration with normal separator (top) and CeO_2_/RGO composite coated separator (bottom) (**b**). The functioning mechanism of CeO_2_/RGO composite coated separator (**c**)

Considering the complex fabrication process of self-standing interlayer, simple and lightweight coating methods were employed in this study. As shown in Fig. 1a, CeO_2_/RGO composite was prepared using a facile polymer pyrolysis followed by hydrothermal technique. The obtained material presented a unique two-dimensional (2D) structure with uniform CeO_2_ nanoparticles anchored on RGO sheets. The CeO_2_/RGO composite was then coated on the traditional commercial separator (Celgard 2400), and the Li/S battery with the modified separator was assembled. The schematic diagrams in Fig. 1b, c revealed that the 2D CeO_2_/RGO composite did not only efficiently inhibit the “shuttle effect” through strong interactions between CeO_2_ and polysulfides, but also enhanced utilization of the active materials due to the fast electron transport of RGO.

## Methods

### Materials and Reagents

Graphene oxide was purchased from The Sixth Element (Changzhou) Materials Technology Company; Ce(NO_3_)_3_·6H_2_O, acrylic acid, and ethanol were purchased from Sinopharm Chemical Reagent Co., Ltd. (Shanghai, China); polyvinylidene fluoride (PVDF) was obtained from Kynar, HSV900; N-methyl-2-pyrrolidone (NMP) and pyrrole (Py) were obtained from Tianjin Guangfu Chemical Reagent; nanosulfur aqueous suspension was purchased from Alfa Chemistry (US Nanomaterials 10 wt%), lithium trifluoromethanesulfony imide (LiTFSI), 1,3-dioxolane (DOL), and 1,2-dimethoxyethane (DME) were obtained from Sigma-Aldrich (Hong Kong, China); Super-P, normal separator (Celgard 2400), Al foil, and lithium mental anode foil was purchased from Li Zhi Yuan battery sales department. Unless otherwise stated, all regents were of analytical grade and used without further purification.

### Preparation of CeO_2_/RGO Composite and Modified Separator

Nanosized CeO_2_ was synthesized using an adapted polymer pyrolysis technique [21]. Firstly, Ce(NO_3_)_3_·6H_2_O and acrylic acid (C_3_H_4_O_2_) at stoichiometric amounts were dissolved in 50 ml deionized (DI) water under constant magnetic stirring at 40 °C to facilitate polymerization. The mixture was then kept stirred until the precursor solution became dry. The obtained product was transferred into a furnace and calcined at 200 °C for 2.5 h in an air atmosphere to yield a polyacrylate salt. The CeO_2_ nanoparticles were finally formed by calcining the polyacrylate salt at 600 °C for 3 h.

A facile hydrothermal technique was used for preparation of CeO_2_/RGO composite. Typically, 4 g graphene oxide was dispersed in DI water to form 40 ml graphene oxide dispersion. After ultrasonication for 1 h, 0.1 g of the as-prepared CeO_2_ nanoparticles were added to the suspension. Next, the mixture was stirred for 2 h to promote the self-assembly of functional groups. Subsequently, the mixture was transferred into an autoclave and heated to 140 °C for 4 h. After drying overnight at 60 °C, the CeO_2_/RGO composite was finally obtained.

The CeO_2_/RGO composite modified separator was prepared by coating the as-prepared composite material onto the surface of normal separator. Typically, 90 wt% of the as-prepared CeO_2_/RGO composite and 10 wt% PVDF in NMP were mixed to form a slurry. After grounding for 40 min, the slurry was coated onto a normal separator by using a spreader with height of 10 mm. Finally, the coated separator was dried at 60 °C in an oven for 6 h.

### Electrode Preparation and Battery Assembly

The fabrication process of the sulfur composite was reported in our previous work [22], which fabricated well-dispersed sulfur anchored on interconnected polypyrrole nanofiber network by mixing PPy and nano-sulfur aqueous suspension via a simple ball-milling followed by a low-temperature heat treatment. The sulfur cathode was prepared by mixing 80 wt% sulfur composite, 10 wt% conductive Super-P, and 10 wt% PVDF binder in NMP then laminated on an aluminum foil at sulfur composite loading around 2.0 mg cm^−2^. Subsequently, the coated foil was dried in vacuum at 60 °C for 6 h. The CR 2032 coin-type cells were assembled using the following components: sulfur cathode, CeO_2_/RGO composite modified separator, Li metal foil anode, and electrolyte containing 1.0 M LiTFSI with 0.1 M LiNO_3_ in mixed dioxolane (DOL) and dimethoxyethane (DME) (1:1 by volume). The amount of electrolyte is around 30 μL.

### Characterization

The morphologies and structures of the samples were observed by scanning electron microscopy (SEM, NovaNano SEM450, FEI) and transmission electron microscopy (TEM, JEM2010F), respectively. The phase composition of CeO_2_/RGO composite was obtained by X-ray diffraction (XRD, Vinci, AXS) with Cu Kα-radiation. The surface functional groups present on the samples were identified by X-ray photoelectron spectroscopy (XPS, ESCALAB250Xi). The Raman spectra were measured using Raman spectroscopy (LabRAM HR Evolution, HORIBA). The specific surface area was examined by the Brunaner-Emmet-Teller (BET) and Barret-Joyner-Halenda (BJH) methods at 77 K (Autosorb iQ, Quantachrome Corporation). The batteries were discharged and charged on a battery test system (BTS-5 V 20 mA, Shenzhen Neware) from 1.5 to 3.0 V at 0.1 C. The electrochemical impedance spectra were collected on an electrochemical workstation (CH1600E) over the frequency range from 0.01–1 MHz.

## Results and Discussion

**Fig. 2:**
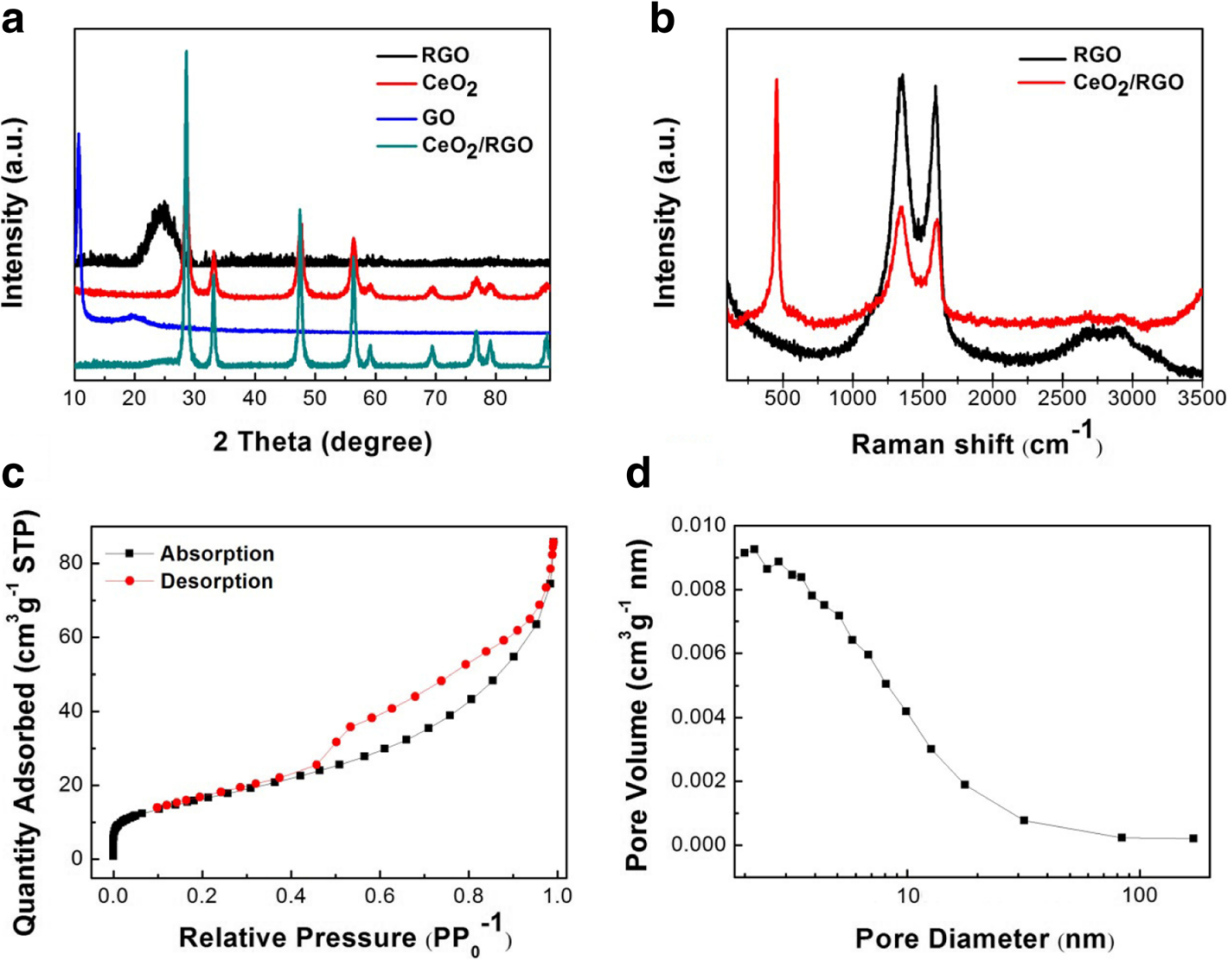
XRD patterns of the samples (**a**). Raman spectra of CeO_2_/RGO composite and RGO (**b**). N_2_ adsorption-desorption isotherm of CeO_2_/RGO composite (**c**). Pore size distribution of CeO_2_/RGO composite (**d**)

Powder XRD was used to identify the phase composition of the as-prepared CeO_2_/RGO composite. The XRD pattern of GO showed a characteristic peak at 2*θ* = 11.5° (Fig. 2a), which can be assigned to (001) plane. After hydrothermal process, a broader peak at 25° associated with (002) plane of carbon had replaced the typical peak of GO, confirming the successful reduction of GO. Diffraction peaks at 2*θ* = 28.5°, 33.0°, 47.5°, 56.3°, 59.0°, 69.4°, 76.7°, 79.0°, and 88.4° corresponding respectively to the (111), (200), (220), (311), (222), (400), (331), (420), and (422) crystalline lattice planes of CeO_2_ were all detected. These peaks agreed well the cubic structure CeO_2_ (JCPDS 65-2975), and the slightly broaden features were attributed to the nanosized nature of CeO_2_ particles. In XRD pattern of CeO_2_/RGO composite, both characteristic peaks of GO and CeO_2_ were observed, indicating that the as-prepared sample was composed of high purity RGO and CeO_2_ phases.

Raman spectroscopy was conducted to detect the disorder degree of the carbon materials by calculating the intensity ratio of D band to G band (I_D_/I_G_). As shown in Fig. 2b, the values of *I*_D_/*I*_G_ were estimated to 0.874 and 0.915 for RGO and CeO_2_/RGO composite, respectively. The increased values suggested the anchoring of CeO_2_ nanoparticles onto RGO sheets. The sharp peak at 455 cm^−1^ was associated with crystalline CeO_2_. Also, no distinct disturbance peak was observed, confirming the successful and efficient synthesis of high purity CeO_2_/RGO composite.

The results of investigation of specific surface area and pore size distribution of as-prepared CeO_2_/RGO composite are shown in Fig. 2c, d, respectively. The N_2_ adsorption/desorption isotherm of CeO_2_/RGO composite shows a large BET-specific surface area of 59.62 m^2^ g^−1^ with the pore volume of 0.1331 cm^3^ g^−1^ and the average pore size of 9.213 nm. Results indicate the porous CeO_2_/RGO composite would benefit the infiltration of electrolyte and transport of electrons.

**Fig. 3:**
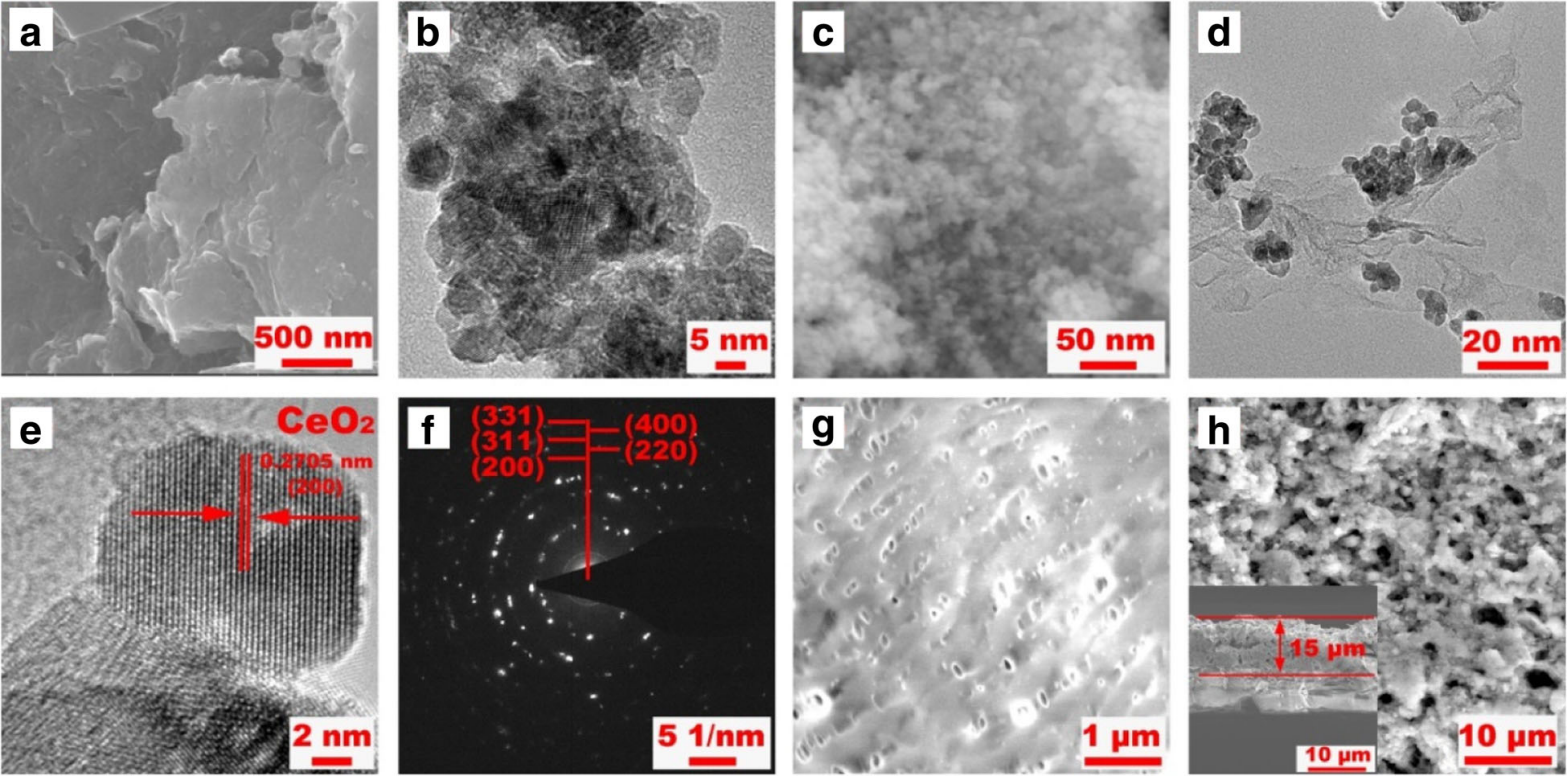
SEM images of pure RGO (**a**), CeO_2_ (**b**), and CeO_2_/RGO composite (**c**). Low-magnification TEM image of CeO_2_/RGO composite (**d**). HRTEM image for CeO_2_/RGO composite (**e**). SAED pattern of CeO_2_/RGO composite (**f**). Top-section SEM image of commercial separator (**g**) and modified separator (**h**). Cross-section SEM image of modified separator (inset to (**h**))

Representative micro-morphologies of RGO, CeO_2_, and CeO_2_/RGO composite are depicted in Fig. 3a–d. Pure RGO sheets showed restacked structures, suffering from reduction in specific surface area. Pure CeO_2_ particles possessed uniform nano sizes but with tendency to agglomerate. Fortunately, the recombination of CeO_2_ and RGO by polymer pyrolysis and hydrothermal methods resulted in unique 2D structure with CeO_2_ nanoparticles well dispersed on RGO sheets. The agglomeration of both RGO sheets and CeO_2_ particles were efficiently inhibited.

High-resolution TEM image of selected area in Fig. 3d is illustrated in Fig. 3e. The lattice spacing of CeO_2_ particles was estimated to 0.2705 nm, well in accordance with the theoretical d-spacing of cubic CeO_2_ (200) plane. The SAED pattern (Fig. 3f) showed homogeneous diffraction rings, confirming the polycrystalline features of the as-prepared CeO_2_/RGO composite. The commercial separator (Celgard 2400) revealed a smooth surface with numerous pores of several micrometers in size (Fig. 3g), whereas sizes of polysulfides (1–1.8 nm) were too small to be hindered by the membrane. By contrast, the holes of pristine separator were completely covered by CeO_2_/RGO composite with a thickness of about 15 μm (Fig. 3h), as well as its inset whose rough surface would benefit the infiltration of electrolyte and transport of electrons. Moreover, the coated CeO_2_/RGO composite can serve as a barrier to block the migration of polysulfides in both physically and chemically.

**Fig. 4:**
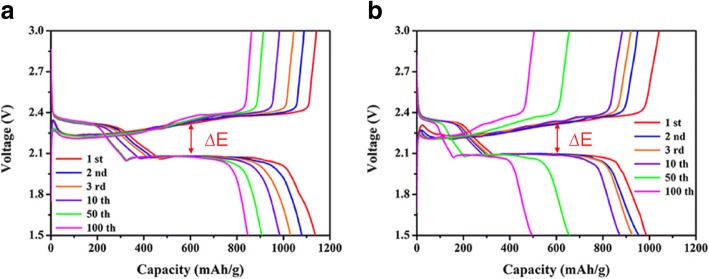
Corresponding charge/discharge voltage-capacity profiles of cells assembled with CeO_2_/RGO composite coated separator (**a**) and pristine separator (**b**)

Charge/discharge voltage-capacity profiles of the cell assembled with CeO_2_/RGO composite modified separator at 0.1 C after different cycling processes are displayed in Fig. 4a. The first discharge plateau was associated with the oxidation processes of S_8_ to Li_2_S_*n*_ (4 ≤ *n* ≤ 8), and the lower plateau corresponded to reduction processes of high-order polysulfides to Li_2_S/Li_2_S_2_ [23]. Stable electrochemical performances were evidently confirmed by the close coincidence curves. The cells assembled with CeO_2_/RGO composite coated separator exhibited overlapping upper discharge plateaus even after 100 cycles, revealing that the modified cell was extremely beneficial for polysulfide inhibition and electrochemical stability. By comparison, the cells with normal separator exhibited shorter upper discharge plateaus accompanied by increased cycling processes. Moreover, the polarization (∆E) of the cells with CeO_2_/RGO composite coated separator (0.224) looked smaller than those assembled with normal separator (0.238). The latter would suggest fast redox reaction kinetics and high system reversibility [24, 25].

**Fig. 5:**
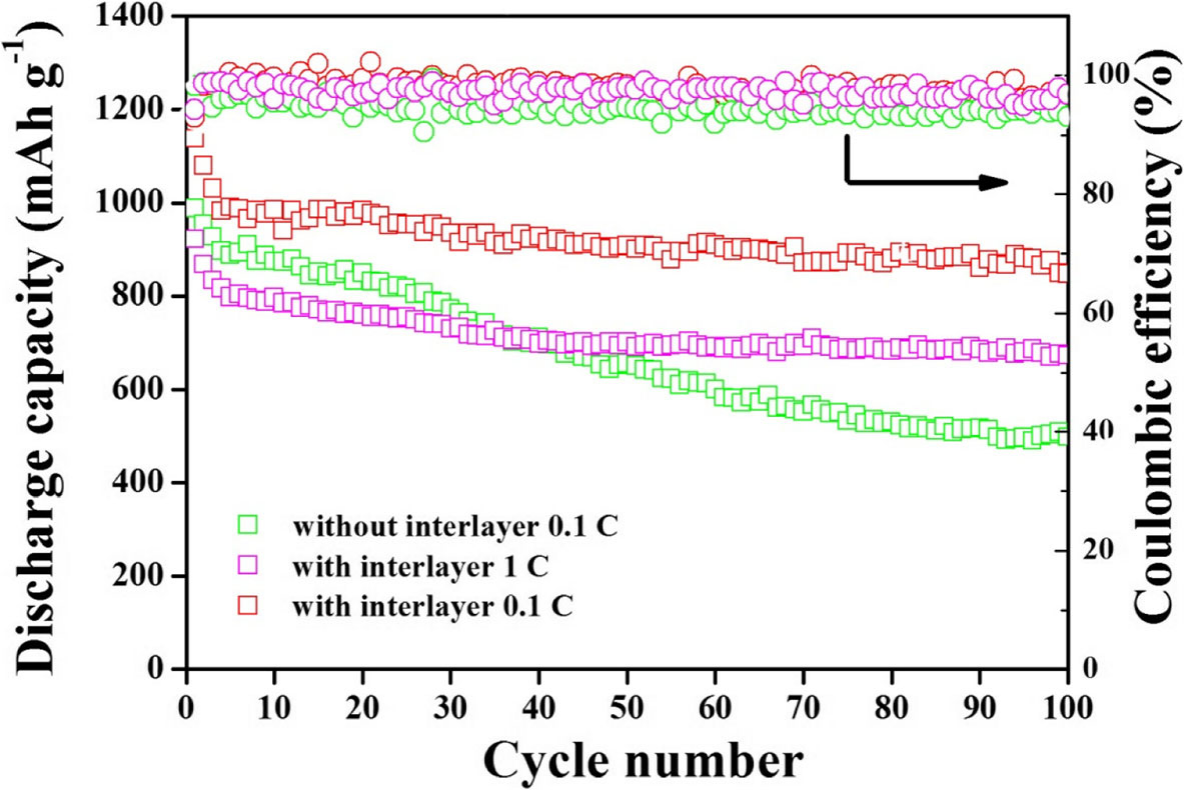
Cycling performance and coulombic efficiency of cells assembled with and without CeO_2_/RGO composite coated separator

The cycling performances of cells assembled with and without CeO_2_/RGO composite modified separator at 0.1 C and 1 C are gathered Fig. 5. At the current rate of 0.1 C, the modified battery achieved a high capacity of 1136 mAh g^−1^ after the 1st cycle and retained a capacity of 886 mAh g^−1^ after 100 cycles with high coulombic efficiency throughout the processes. These values were superior to that of the cell assembled with normal separator (992 mAh g^−1^ and 501 mAh g^−1^, respectively), suggesting the key role played by the functional separator. In addition, when the current rate increased to 1 C, the modified cells can also delivered an outstanding initial capacity of 917 mAh g^−1^ and maintained 72.9% of its initial capacity as well as high coulombic efficiency throughout the processes. The well-designed structure would not only allow better transport of electrons by contribute to superior electrical conductivity of RGO. Also, the shuttling of polysulfides could efficiently be impeded by the strong chemical bond between CeO_2_ and sulfur-related species.

**Fig. 6:**
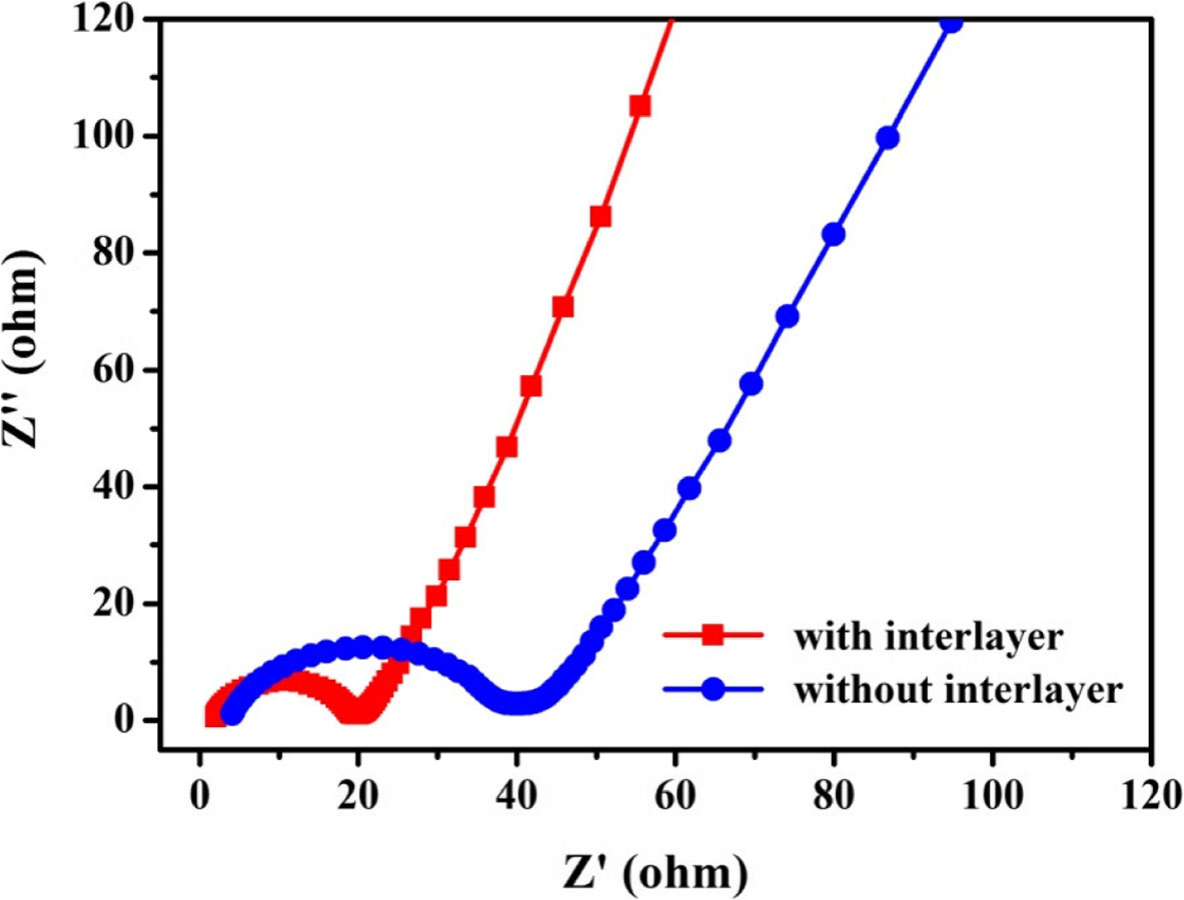
Nyquist plots for cells assembled with and without CeO_2_/RGO composite coated separator

The Nyquist plots of the cells assembled with and without CeO_2_/RGO composite-modified separator were first obtained then fitted with an equivalent circuit model. As shown in Fig. 6, both cells exhibited depressed semicircle in high-frequency region and inclined line at low frequencies. These would correspond to charge-transfer resistance (*R*_CT_) for sulfur cathode and Li-ion diffusion or so-called Warburg impedance, respectively [26, 27]. The smaller semicircle represented moderate *R*_CT_ value of the modified cell, which mainly attributed to the efficiently suppressed shuttling of polysulfides by CeO_2_ nanoparticles and superior electron transport of RGO. Moreover, the CeO_2_/RGO composite would improve the electrochemical contact and maximize the utilization of active materials. The larger slope of Warburg impedance in modified cells suggested shortened diffusion of Li ions.

**Fig. 7:**
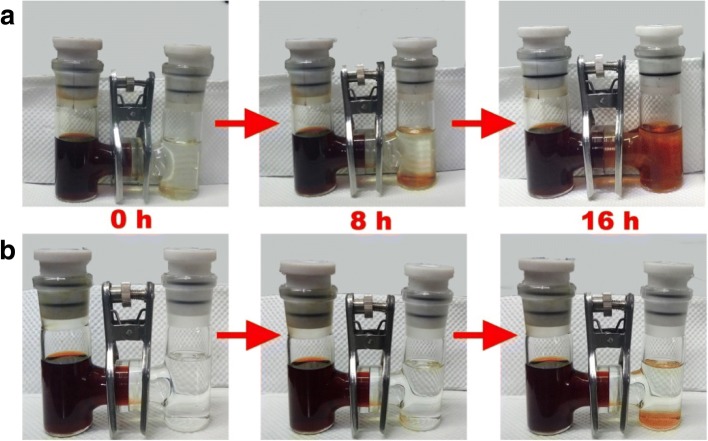
Photographs of H-type glass cells assembled with pristine separator (**a**) and CeO_2_/RGO composite coated separator (**b**)

To gain a better understanding about contributions of CeO_2_/RGO composite-coated separator in impeding the shuttle of sulfur-related species, H-type glass cells were introduced and tested. As displayed in Fig. 7, the dark brown solution in the left side was composed of DOL/DME with 0.05 M Li_2_S_6_ as an additive. The right side solution contained pure DOL/DME. Li_2_S_6_ would spontaneously diffuse through the membrane from high to low concentration, which can be reflected by changes in color [28, 29]. In cells with normal separator (Fig. 7a), the color of the right cell changed evidently over time to become dark brown after 16 h, confirming that traditional commercial separator was unable to hinder the diffusion of polysulfide. By comparison, in cells with CeO_2_/RGO composite coated separator (Fig. 7b), no distinct color change took place over time, suggesting the shuttling of polysulfide was inhibited by CeO_2_/RGO composite modified separator.

**Fig. 8:**
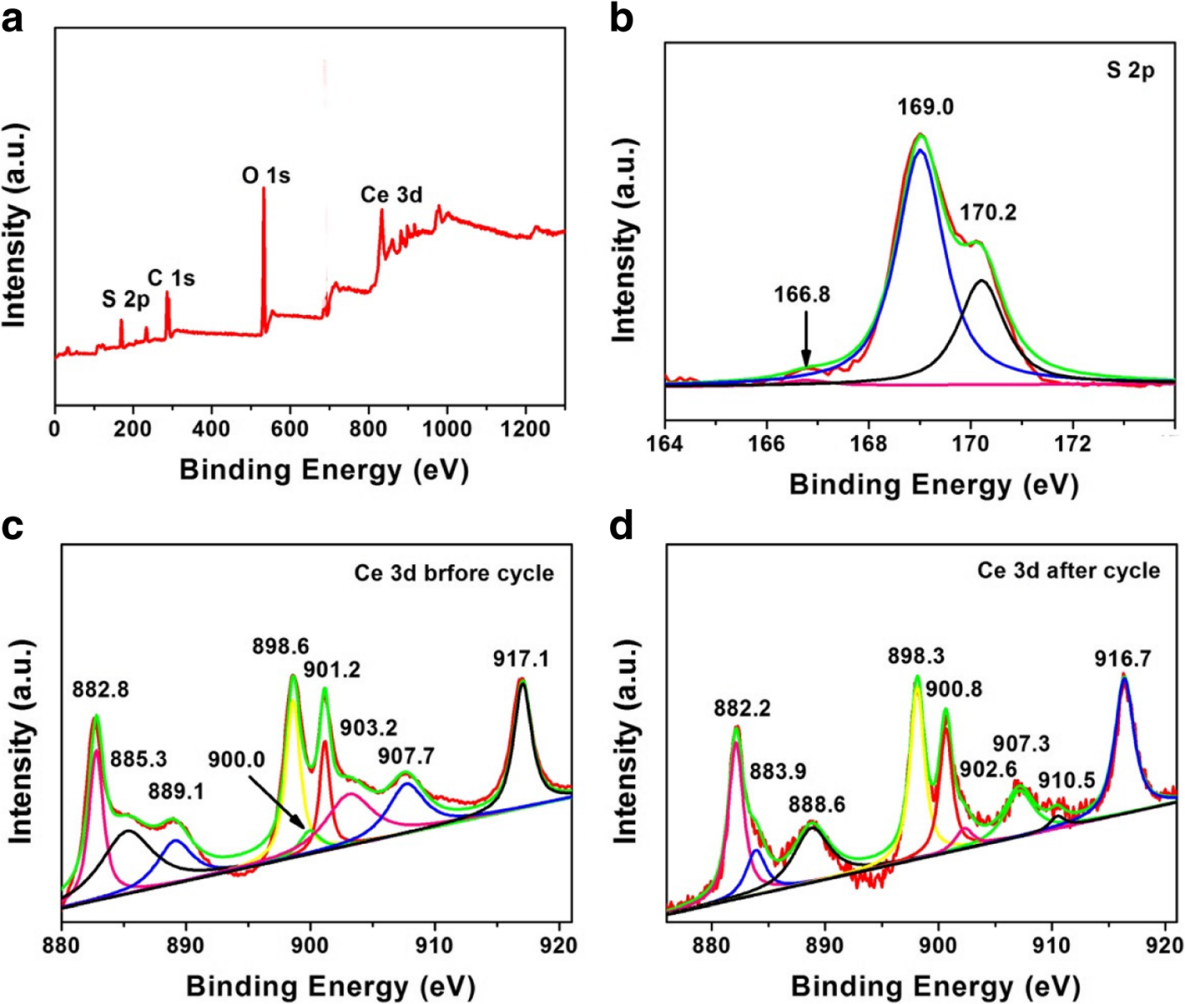
XPS spectra of CeO_2_/RGO composite after cycling: survey spectrum (**a**) and S 2*p* (**b**). XPS spectra of CeO_2_/RGO composite: Ce 3*d* before (**c**) and after cycling (**d**)

XPS was used to confirm the existence of interactions between CeO_2_ and sulfur-related species. The elemental composition and valence states of CeO_2_/RGO composite after cycling are displayed in Fig. 8a. Four elements (C, O, Ce, and S) were detected. The peak in S 2*p* spectrum of CeO_2_/RGO composite after cycling can be fitted by three parts (Fig. 8b). The peak observed at 166.8 eV was assigned to S–O, and the peaks at 169.0 and 170.2 eV might be caused by metal-SO_4_^2−^ species. The Ce 3*d* spectrum of CeO_2_/RGO composite after cycling revealed peaks at binding energies of 882.8, 885.3, 889.1, and 898.6 eV (Fig. 8c), corresponding to CeO_2_ 3*d* 5/2. The peak at 885.3 eV can be attributed to CeO_2_ 3*d* 5/2. The peaks located at 901.2, 907.7, and 917.1 eV were associated with CeO_2_ 3*d* 3/2. The peaks of CeO_2_/RGO composite-coated separator after cycling appeared slightly shifted to negative values (Fig. 8d). This indicated absorption of sulfur-related species by Ce–S bonding [30], corresponding to S 2*p* spectrum of CeO_2_/RGO composite after cycling.

## Conclusions

Polymer pyrolysis and hydrothermal method were employed as facile and efficient ways to prepare CeO_2_/RGO composite with superior structure, where ultrafine CeO_2_ nanoparticles were anchored on RGO sheets. The chemical suppression of the shuttling effect of polysulfides for CeO_2_ was confirmed by XPS after electrochemical processes. The performance of Li/S battery was significantly enhanced due to the cooperation of RGO and CeO_2_. A high initial capacity of 1136 mAh g^−1^ was obtained at 0.1 C with about 75.7% capacity retention after 100 cycles. The coulombic efficiency of the cell with CeO_2_/RGO composite-coated separator was also higher than values obtained by traditional commercial separators.

## Abbreviations

CeO_2_: Cerium oxide
DME: 1,2-Dimethoxyethane
DOL: 1,3-Dioxolane
GO: Graphene oxide
HRTEM: High-resolution transmission electron microscope
Li/S: Lithium/sulfur
LiTFSI: Lithium bis(trifluoromethanesulfonyl)imide
NMP: N-methyl-2-pyrrolidene
PVDF: Polyvinylidene fluoride
R_CT_: Charge-transfer resistance
RGO: Reduced graphene oxide
SAED: Selected area electron diffraction
SEM: Scanning electron microscope
TEM: Transmission electron microscope
XPS: X-ray photoelectron spectroscopy
XRD: X-ray diffraction
